# The Role of Long-Term Physical Activity in Relation to Cancer-Related Health Outcomes: A 12-Month Follow-up of the Phys-Can RCT

**DOI:** 10.1177/15347354231178869

**Published:** 2023-06-26

**Authors:** Anne-Sophie Mazzoni, Ann Christin Helgesen Bjørke, Andreas Stenling, Sussanne Börjeson, Katarina Sjövall, Sveinung Berntsen, Ingrid Demmelmaier, Karin Nordin

**Affiliations:** 1 Uppsala University, Uppsala, Sweden; 2 University of Agder, Kristiansand, Norway; 3 Umeå University, Umeå, Sweden; 4 Linköping University, Linköping, Sweden; 5 Kristianstad University, Kristianstad, Sweden

**Keywords:** physical activity patterns, correlates, cancer survivorship, health outcomes, accelerometer

## Abstract

**Purpose:**

While moderate-to-vigorous intensity physical activity (MVPA) is associated with various health improvements shortly after completion of exercise interventions, it remains unclear which health benefits can be expected when MVPA levels are maintained in the long term in cancer survivors. We aimed to assess the associations of (1) MVPA level at 12-month follow-up and (2) long-term MVPA patterns (from immediately post-intervention to 12-month follow-up) with different cancer-related health outcomes.

**Methods:**

In the Physical training and Cancer (Phys-Can) RCT, 577 participants diagnosed with breast (78%), prostate (19%), or colorectal (3%) cancer were randomized to 6 months of exercise during curative cancer treatment. Accelerometer-assessed physical activity and outcome data (ie, cancer-related fatigue, health-related quality of life [HRQoL], anxiety and depression, functioning in daily life, cardiorespiratory fitness, sedentary time and sleep) were collected immediately post-intervention and at 12-month follow-up. Based on the sample’s median of MVPA immediately post-intervention (65 minutes/day) and the changes between the 2 measurement points, 4 categories with different long-term MVPA patterns were created: High & Increasing, High & Decreasing, Low & Increasing, and Low & Decreasing. Multiple linear regression analyses were performed for the analyses.

**Results:**

A total of 353 participants were included in the analyses. At 12-month follow-up, a higher MVPA level was significantly associated with lower fatigue in 3 domains (general fatigue [β = −.33], physical fatigue [β = −.53] and reduced activity [β = −.37]), higher cardiorespiratory fitness (β = .34) and less sedentary time (β = −.35). For long-term MVPA patterns, compared to the participants in the “Low & Decreasing” category, those in the “High & Increasing” category reported significantly lower fatigue in 3 domains (general fatigue [β = −1.77], physical fatigue [β = −3.36] and reduced activity [β = −1.58]), higher HRQoL (β = 6.84) and had less sedentary time (β = −1.23).

**Conclusion:**

Our results suggest that long-term physical activity is essential for improving health outcomes post-intervention in cancer survivors. Cancer survivors, including those who reach recommended MVPA levels, should be encouraged to maintain or increase MVPA post-intervention for additional health benefits.

**Trial registration:**

NCT02473003 (10/10/2014)

## Introduction

Regular physical activity has proven safe and beneficial in cancer survivors.^1^ Systematic reviews have demonstrated positive effects of physical activity on many cancer-related health outcomes such as cancer-related fatigue,^2^ depression,^3^ anxiety,^4^ health-related quality of life (HRQoL)^5^ and physical fitness^6^ during and after cancer treatment. Cancer survivors have much to gain from regular physical activity and are therefore recommended to engage in at least 150 minutes/week of moderate-intensity physical activity, or 75 minutes/week of vigorous-intensity physical activity, or an equivalent combination of the 2 (ie, moderate-to-vigorous-intensity physical activity, MVPA).^7^ Nevertheless, it is challenging for many cancer survivors to engage in physical activity. The majority tend to reduce their level of physical activity during the years following a cancer diagnosis^8^ and are less physically active than those without a history of cancer.^9^ Moreover, cancer survivors also struggle to maintain their level of physical activity after participating in an exercise intervention.^10-12^ Given its potential health benefits, long-term physical activity (≥12 months post-intervention^13^) is a research area of particular importance in exercise oncology but has been poorly studied.^13,14^ Indeed, previous research has mainly focused on physical activity shortly after completion of exercise interventions (≤6 months post-intervention), indicating that maintaining physical activity levels during this period is crucial for cancer survivors to achieve sustained health benefits (eg, reduced cancer-related fatigue, improved cardiorespiratory fitness and HRQoL).^15,16^ However, few studies have examined such associations in the long term (≥12 months post-intervention).^17-19^ Consequently, it remains unclear which health benefits can be expected in cancer survivors when physical activity levels are maintained in the long term after completion of exercise interventions. Additionally, physical activity is rarely objectively assessed in this population,^14^ limiting detailed analyses and a reliable interpretation of study results. Such investigations are needed to optimize future exercise interventions aiming at improving physical activity and cancer-related health outcomes in the long term in cancer survivors.

We recently reported the results from a 6-month exercise intervention study with a 2 × 2 factorial design, the Physical Training and Cancer randomized controlled trial (Phys-Can RCT).^20-22^ In the above-mentioned study, we examined the effects of high (HI) versus low-to-moderate-intensity (LMI) exercise *with* or *without* additional behavior change support (BCS; eg, goal-setting and self-monitoring) on different cancer-related health outcomes^20^ and on exercise adherence^21^ in patients undergoing curative cancer treatment. Our results indicated small differences between groups at the end of the intervention in physical fatigue, muscle strength, and cardiorespiratory fitness (favoring HI exercise), but no effects on the other outcomes were found.^20^ Moreover, no effect of the additional BCS was found on exercise adherence during the intervention.^21^ However, at 12-month follow-up, the groups randomized to additional BCS maintained their physical activity level to a greater extent compared to the groups without BCS, indicating a delayed effect of the additional BCS on physical activity.^22^ Given this delayed effect, it is of interest to examine if there are differences in other cancer-related health outcomes at 12-month follow-up between participants with different MVPA levels and patterns. Assessing both physical activity at a specific time point (ie, MVPA level at 12-month follow-up) and physical activity over time (ie, long-term MVPA patterns from immediately post-intervention to 12-month follow-up) enables a detailed description and analysis of physical activity.^23^

In the present study, we investigated MVPA in cancer survivors who previously participated in an exercise intervention during curative cancer treatment. The aims were to assess the associations of (1) MVPA level at 12-month follow-up and (2) long-term MVPA patterns (from immediately post-intervention to 12-month follow-up) with different cancer-related health outcomes (ie, cancer-related fatigue, HRQoL, anxiety/depression, function in daily life, cardiorespiratory fitness, sedentary time and sleep). We hypothesized that higher MVPA levels at 12-month follow-up and increased MVPA patterns after the end of an exercise intervention would be associated with improved health outcomes.

## Methods

### Settings and Participants

Data included in the present study were collected as part of the Phys-Can RCT, a multicentre trial (NCT02473003).^20,24^ The Phys-Can RCT was approved by the Regional Ethical Review Board in Uppsala (Dnr 2014/249).^20,24^ All participants provided written informed consent. The recruitment process and design have previously been described in detail.^20^ Briefly, eligible patients were aged 18 years or older, newly diagnosed with breast, colorectal or prostate cancer and scheduled to undergo curative treatment as follows: (1) (neo-)adjuvant chemotherapy and/or adjuvant radiation therapy and/or adjuvant endocrine therapy *for patients with breast cancer*, (2) adjuvant radiation therapy with or without (neo-)adjuvant endocrine therapy *for patients with prostate cancer*, and (3) adjuvant chemotherapy with or without neoadjuvant radiation therapy *for patients with colorectal cancer*. Patients were excluded if they were diagnosed with stage IIIb-IV breast cancer, suffered from cognitive dysfunction (eg, dementia or serious mental illness), physical impairments and/or other diseases (eg cardiovascular or lung diseases) that could affect their ability to perform physical activity and exercise. The recruitment took place at university hospitals in 3 cities in Sweden (Lund/Malmö, Linköping and Uppsala) between March 2015 and April 2018. Randomization was stratified by cancer diagnosis and city. Participants were randomly assigned to 1 of 4 intervention groups: HI versus LMI exercise *with* or *without* additional BCS. Participants included in the present study were those with MVPA data available at 12-month follow-up (12 months post-intervention).

### Intervention

#### Exercise program

The exercise program has been described previously in detail.^20^ Briefly, participants performed resistance training and endurance training for 6 months while undergoing curative cancer treatment. The resistance training was group-based and supervised by coaches, specifically trained for the study (physiotherapists and personal trainers). The program consisted of a total of 6 machine-based exercises and was performed twice a week. Participants alternated between 3 × 6 repetitions maximum (RM) and 3 × 10 RM in the HI groups, and 3 × 12 repetitions at 50% of 6 RM and 3 × 20 repetitions at 50% of 10 RM in the LMI groups. The endurance training was home-based and consisted of twice-weekly interval training (20-40 minutes/session) at 80–90% of heart rate reserve (HRR) in the HI groups, and 150 minutes weekly continuous-based exercise at 40–50% of HRR in the LMI groups.

#### Additional BCS

Additional BCS used in the Phys-Can RCT have been described previously in detail.^20^ Briefly, participants randomized to receive additional BCS were guided by coaches to use weekly goal-setting, review of behavioral goal, self-monitoring, action planning, problem solving, and follow-up prompts. The additional BCS was provided face-to-face, jointly with the resistance training sessions on a maximum of 9 occasions, except for self-monitoring which was performed by the participants after each exercise session. Weekly meetings were offered during the first month to provide participants with a gradual introduction to the use of the additional BCS. For example, the coaches assisted participants in formulating concrete and realistic goals as well as exercise plans specifying when, where and how to perform home-based endurance training. After the first month, those meetings were held every 4 to 6 weeks, depending on individual needs. The coaches used printed sheets during each meeting to make notes about what was decided (goal-setting, exercise planning). Those notes were then discussed during the next meeting, and if participants did not manage to exercise according to their planning, strategies to overcome barriers to exercise were identified and adjustments were made. Participants also developed an individual written plan for relapse prevention at the end of the exercise intervention, including goal-setting and coping planning. This plan was individually followed up and revised at 3 and 9 months post-exercise intervention in face-to-face or telephone meetings with the coaches.

### Measures

#### Physical activity

Two physical activity measures, MVPA level at 12-month follow-up and long-term MVPA patterns (from immediately post-intervention to 12-month follow-up) were assessed with SenseWear Armband mini (SWA) immediately post-intervention and at 12-month follow-up. The SWA is a monitor combining a tri-axial accelerometer with heat/skin sensors, and has previously been validated in healthy adults^25,26^ and in cancer survivors.^27^ At each measurement point, participants were asked to wear the SWA 24 hours a day for 7 consecutive days. To reflect 1 week of MVPA, data from the SWA were included in the analyses if the SWA was worn for at least 4 days,^28^ including one weekend day^29^ with a wear time of at least 80% per day.^30^ The Professional 8.1 Software was used to provide SWA wear time and minutes spent in different levels of Metabolic Equivalent Task values (METs). MVPA was determined using the established cut-point of ≥3.0 METs.^31^ Daily time spent in MVPA levels was calculated by summing minutes for each valid day, where the criterion for MVPA was met.

The first physical activity measure, MVPA level at 12-month follow-up, was assessed as *mean minutes/day in MVPA at 12-month follow-up* and was converted to units of 30 minutes (eg, 15 minutes = 0.5, 30 minutes = 1 and 60 minutes = 2) to facilitate the interpretation of the results. The second physical activity measure, long-term MVPA patterns, was created based on the study sample’s median of MVPA immediately post-intervention (ie, high MVPA level ≥ median and low MVPA level <median) and the changes of MVPA level between immediately post-intervention and 12-month follow-up (increased or decreased). Participants were then categorized as either (1) “High & Increasing” (participants with high MVPA level immediately post-intervention and increased MVPA at 12-month follow-up), (2) “High & Decreasing” (participants with high MVPA level immediately post-intervention and decreased MVPA at 12-month follow-up), (3) “Low & Increasing” (participants with low MVPA level immediately post-intervention and increased MVPA at 12-month follow-up) or (4) “Low & Decreasing” (participants with low MVPA level immediately post-intervention and decreased MVPA at 12-month follow-up).

#### Cancer-related health outcomes

All cancer-related health outcomes were assessed at 12-month follow-up. Cancer-related fatigue was assessed with the Multidimensional Fatigue Inventory questionnaire (MFI-20, range 4-20),^32^ and consists of 20 items divided into 5 subscales (domains): general fatigue, physical fatigue, mental fatigue, reduced motivation and reduced activity. HRQoL was assessed with the subscale of European Organisation for Research and Treatment of Cancer quality of life questionnaire (EORTC QLQ-C30, range 0-100).^33^ Anxiety and depression were assessed with Hospital Anxiety and Depression Scale (HADS, range 0-21).^34^ Functioning in daily life was assessed with World Health Organization Disability Assessment Schedule (WHODAS) 2.0 Work subscale (range 0-16) and Social Participation subscale (range 0-32).^35^ Cardiorespiratory fitness was measured as maximal oxygen uptake (VO_2_max, mL/kg/min) during walking/running on a treadmill to exhaustion using a modified Balke-protocol.^36^ Finally, sedentary time and sleep (mean hours per day) were measured with the SWA. Sedentary time was considered to be time spent at 1.5 METs or less during waking hours^37^ according to the Professional 8.1 Software. SWA has previously been validated for assessing sedentary time^38^ and sleep duration^39^ in adult populations.

### Statistical Analyses

Descriptive characteristics are presented as mean and standard deviation (*SD*) for continuous variables and proportions as number (*n*) and percentage (%) for categorical variables. Differences in baseline characteristics between participants in the different pattern categories as well as differences between participants included in the analysis and those who were lost to follow-up were examined using one-way ANOVA or independent *t*-test for continuous variables and Chi^2^ test for nominal variables when appropriate.

Multiple linear regression was performed to examine the associations of (1) MVPA level at 12-month follow-up and (2) long-term MVPA patterns with each cancer-related health outcome, respectively. For the analyses with long-term MVPA patterns as independent variable, the variable was included in models using dummy-coding (High & Increasing/ High & Decreasing/ Low & Increasing/ Low & Decreasing) and the category “Low & Decreasing” was used as reference in the analyses to test the hypothesis that high and/or increased MVPA after the end of an exercise intervention would be associated with improved health outcomes compared to those remaining less physically active. All cancer-related health outcomes were treated as continuous variables. All models included immediately post-intervention value of the health outcome to increase precision and were adjusted for age, gender, and cancer treatment. The results are presented as unstandardized regression coefficients (β) with 95% Confidence Intervals (95% CI). The unstandardized coefficients can be interpreted as the mean change in the outcome (1) per 30 minutes/day increase in MVPA for the analyses with MVPA level at 12-month follow-up and (2) when comparing the group “High & Increasing,” “High & Decreasing” or “Low & Increasing” with the reference group “Low & Decreasing” for the analyses with long-term MVPA patterns.

Missing data were <10% in all cancer-related health outcomes, except for functioning in daily life (13% missing) and cardiorespiratory fitness (24% missing). Multiple imputation was used to handle missing data in the cancer-related health outcomes.^40^ Age, cancer treatment and baseline values of the cancer-related health outcomes were used as auxiliary variables to inform imputed values. Twenty different datasets were created and pooled estimates were calculated using Rubin’s rules.^40^ Analyses were performed with and without imputed data. As the results were similar, the regression analyses with imputation are therefore presented. All statistical tests were 2-sided and considered statistically significant if *P* < .05. Multiple imputation and statistical tests were performed using the Statistical Package for the Social Sciences (SPSS, v.27).

## Results

### Participants

Of the 577 participants included in the Phys-Can RCT, 353 (61%) had available SWA data at 12-month follow-up (12 months post-intervention) and were included in the present study. Of those participants, 316 (90%) also had available SWA data at 6 months (immediately post-intervention) and were categorized as “High & Increasing” (n = 46), “High & Decreasing” (n = 112), “Low & Increasing” (n = 91) or “Low & Decreasing” (n = 67).

Participants’ baseline characteristics are presented in Table 1. Their mean age was 59 years (*SD* 12). The majority were diagnosed with breast cancer (78%) and received chemotherapy as primary (neo-) adjuvant treatment (53%). Differences in baseline characteristics were observed between the follow-up participants (Table 1). For example, participants in the “Low & Increasing” category were significantly older (*P* = .04), less physically active and had a higher body mass index (*P* < .001) compared to those in the “High & Increasing” and “High & Decreasing” categories. Furthermore, the 224 participants (39%) lost to follow-up (had no available SWA data at 12-month follow-up) significantly differed from the follow-up population in having a lower education level (*P* = .01), a higher body mass index (*P* = .01) and poorer aerobic exercise habits (*P* = .02) at baseline (Table 1). Descriptive data for physical activity and cancer-related health outcome measures post-intervention are presented in Supplemental Tables 1 and 2, respectively.

**Table 1.** Baseline Characteristics of Follow-Up Participants and Lost to Follow-Up Participants in the Phys-Can RCT.

	Follow-up participants (n = 353)					Lost to follow-up participants (n = 224)
	All (n = 353)	Long-term MVPA patterns^a^ (n = 316)				
		High & Increasing (n = 46)	High & Decreasing (n = 112)	Low & Increasing(n = 91)	Low & Decreasing(n = 67)	
Age, mean (*SD*)	59 (12)	56 (12)	58 (12)	62 (12)	59 (11)	58 (12)
Women, n (%)	280 (79)	34 (74)	80 (71)	70 (77)	64 (96)	185 (83)
Living with partner, n (%)	268 (78)	31 (69)	84 (77)	76 (85)	51 (76)	163 (78)
High education (university), n (%)	229 (65)	32 (70)	71 (63)	58 (64)	46 (69)	117 (52)
Occupation, n (%)						
Working^b^	116 (34)	20 (43)	36 (33)	33 (37)	20 (31)	57 (27)
On sick leave	118 (34)	16 (35)	39 (36)	23 (26)	23 (36)	78 (37)
Retired	109 (32)	10 (22)	33 (31)	33 (37)	21 (33)	73 (35)
Comorbidities ≥1, *n* (%)	181 (57)	21 (50)	49 (51)	56 (66)	33 (56)	112 (60)
Weight status, n (%)						
Normal weight, BMI 18-24.9 kg/m^2^	181 (53)	30 (71)	67 (62)	38 (43)	26 (39)	86 (43)
Pre-obese, BMI 25-29.9 kg/m^2^	117 (34)	10 (24)	36 (33)	33 (37)	24 (36)	71 (35)
Obese, BMI > 29.9 kg/m^2^	43 (13)	2 (5)	6 (6)	18 (20)	16 (24)	44 (22)
MVPA, median min/day (IQR)	65 (58)	99 (44)	87 (70)	59 (40)	44 (43)	51 (54)
Current exercise habits, *n* (%)						
Regular aerobic exercise	122 (41)	19 (49)	49 (45)	24 (31)	14 (25)	65 (35)
Regular resistance exercise	68 (23)	17 (45)	24 (27)	10 (14)	7 (13)	35 (19)
Diagnosis, n (%)						
Breast cancer	276 (78)	34 (74)	79 (71)	69 (76)	62 (93)	181 (81)
Prostate cancer	65 (19)	9 (20)	31 (28)	19 (21)	2 (3)	32 (14)
Colorectal cancer	12 (3)	3 (7)	2 (2)	3 (3)	3 (5)	11 (5)
Cancer stage^c^, n (%)						
Stage 0 (In situ)	9 (3)	2 (5)	3 (3)	0 (0)	3 (5)	7 (5)
Stage 1	157 (49)	21 (49)	39 (40)	48 (59)	33 (52)	78 (51)
Stage 2	124 (39)	17 (39)	44 (45)	27 (33)	24 (38)	56 (37)
Stage 3	30 (9)	3 (7)	11 (11)	7 (8)	3 (5)	11 (7)
Primary (neo-)adjuvant treatment, n (%)						
Chemotherapy	188 (53)	30 (65)	56 (50)	42 (46)	34 (51)	129 (58)
Radiation therapy	116 (33)	14 (31)	34 (30)	34 (37)	27 (40)	69 (31)
Endocrine therapy	49 (14)	2 (4)	22 (20)	15 (17)	6 (9)	26 (12)

*Abbreviations*: Phys-Can, Physical Training and Cancer; RCT, randomized controlled trial; SD, standard deviation; BMI, body mass index; MVPA, moderate-to-vigorous intensity physical activity; IQR, interquartile range.

*Note*: n’s do not all sum to total due to missing data; % is of those with available data.

a Participants’ level of moderate-to-vigorous intensity physical activity at 12-month follow-up in relation to immediately post-intervention levels, 4 categories with different long-term MVPA patterns were created :“High & Increasing” (participants with high MVPA level immediately post-intervention and increased MVPA at 12-month follow-up), “ High & Decreasing” (participants with high MVPA level immediately post-intervention and decreased MVPA at 12-month follow-up), “Low & Increasing ” (participants with low MVPA level immediately post-intervention and increased MVPA at 12-month follow-up) and “Low & Decreasing” (participants with low MVPA level immediately post-intervention and decreased MVPA at 12-month follow-up).

b Full-time and part-time, not on any sick leave.

c According to the seventh edition of the Tumour-Node-Metastasis (TNM) clinical classification.^41^

### Associations of MVPA Level With Cancer-Related Health Outcomes

The associations of MVPA level at 12-month follow-up with cancer-related health outcomes are presented in Table 2.

**Table 2.** Associations of MVPA Level at 12-month Follow-Up with Cancer-Related Health Outcomes, Presented as Mean Change in the Outcomes per 30 minutes/day Increase in MVPA and 95% Confidence Intervals (n = 353).

Outcome	β (95% CI)	*p*-Value
Cancer-related fatigue (MFI, 4-20)^a^		
General fatigue	**−.33 (−0.55 to −0.10)**	**.005**
Physical fatigue	**−.53 (−0.79 to −0.28)**	<**.001**
Reduced activity	**−.37 (−0.58 to −0.16)**	**.001**
Reduced motivation	**−**.11 (**−**0.30 to 0.08)	.240
Mental fatigue	**−**.06 (**−**0.26 to 0.14)	.555
HRQoL (EORTC QLQ-C30, 0-100)^b^	.22 (−0.83 to 1.28)	.678
Anxiety and depression (HADS, 0-21)^a^		
Anxiety	**−**.08 ( **−**0.25 to 0.08)	.322
Depression	**−**.12 (**−**0.26 to 0.03)	.127
Functioning in daily life (WHODAS) ^a^		
Work subscale (0-16)^c^	.13 (**−**0.18 to 0.45)	.411
Social Participation subscale (0-32)	**−**.20 (**−**0.46 to 0.08)	.159
Cardiorespiratory fitness (VO2max, mL/kg/min)^b^	**.34 (0.06 to 0.62)**	**.016**
Sedentary time (SWA, h/d)^a^	**−.35 (−0.44 to −0.27)**	<**.001**
Sleep (SWA, h/d)	.02 (**−**0.04 to 0.07)	.623

*Abbreviations*: MVPA, moderate-to-vigorous intensity physical activity; β, unstandardized regression coefficients; MFI, Multidimensional Fatigue Inventory; EORTC QLQ-C30, European Organisation for Research and Treatment of Cancer; HADS, Hospital Anxiety and Depression scale; WHODAS, World Health Organization Disability Assessment Schedule; VO2max, maximal volume of oxygen uptake; SWA, SenseWear Armband mini.

*Note:* All models included immediately post-intervention value of the outcome to increase precision and were adjusted for age, gender and cancer treatment. Bold indicates *p*-value < .05.

a Higher scores indicate worse outcome.

b Higher scores indicate better outcome.

c For participants who reported working.

A daily increase of 30 minutes MVPA at 12-month follow-up was significantly associated with lower fatigue in 3 domains: general fatigue (β = −.33, 95% CI [− 0.55 to −0.10], P = .005), physical fatigue (β = −.53, 95% CI [−0.79 to −0.28], P < .001) and reduced activity (β = −.37, 95% CI [−0.58 to −0.16], P = .001).

Additionally, a daily increase of 30 minutes MVPA at 12-month follow-up was significantly associated with higher cardiorespiratory fitness (β = .34, 95% CI [0.06-0.62], *P* = .016) and less sedentary time (β = −35, 95% CI [−0.44 to −0.27], *P* < .001) (Table 2).

### Associations of Long-Term MVPA Patterns With Cancer-Related Health Outcomes

The associations of long-term MVPA patterns from immediately post-intervention to 12-month follow-up with cancer-related health outcomes are presented in Table 3.

**Table 3.** Associations of Long-Term MVPA Patterns from Immediately Post-Intervention to 12-month Follow-Up with Cancer-Related Health Outcomes, Presented as Mean Change in the Outcomes and 95% Confidence Intervals (n = 316).

Outcome	High & Increasing vs Low & Decreasing		High & Decreasing vs Low & Decreasing		Low & Increasing vs Low & Decreasing	
	β (95% CI)	*p*-Value	β (95% CI)	*p*-Value	β (95% CI)	*p*-Value
Cancer-related fatigue (MFI, 4-20)^a^						
General fatigue	**−1.77 (−3.08 to −0.46)**	**.008**	−0.86 (−1.92 to 0.22)	.119	−0.32 (−1.39 to 0.76)	.564
Physical fatigue	**−3.36 (−4.83 to −1.89)**	<**.001**	**−1.90 (−3.09 to −0.71)**	**.002**	−0.46 (−1.66 to 0.74)	.454
Reduced activity	**−1.58 (−2.82 to −0.34)**	**.012**	−0.39 (-1.39 to 0.61)	.44	−0.01 (−1.03 to 1.01)	.981
Reduced motivation	−0.91 (-1.97 to 0.14)	.089	−0.71 (-1.57 to 0.14)	.102	−0.05 (−0.82 to 0.92)	.913
Mental fatigue	−0.33 (−1.48 to 0.82)	.575	−0.61 (−1.54 to 0.31)	.194	−0.11 (−1.06 to 0.84)	.824
HRQoL (EORTC QLQ-C30, 0-100)^b^	**6.84 (0.84-12.84)**	**.026**	4.64 (-0.25 to 9.52)	.063	3.78 (−1.20 to 8.76)	.137
Anxiety and depression (HADS, 0-21)^a^						
Anxiety	−0.18 (−1.16 to 0.83)	.727	−0.04 (−0.83 to 0.76)	.930	−0.22 (−1.04 to 0.60)	.601
Depression	−0.44 (−1.31 to 0.42)	.320	−0.17 (−0.86 to 0.53)	.642	0.40 (−0.31 to 1.11)	.270
Functioning in daily life (WHODAS)^a^						
Work subscale (0-16)^c^	0.73 (−1.25 to 2.71)	.470	−0.63 (−2.05 to 0.78)	.380	−0.32 (−1.83 to 1.19)	.679
Social Participation subscale (0-32)	−1.09 (−2.66 to 0.47)	.170	**−1.43 (−2.70 to −0.16)**	**.027**	−0.69 (−1.99 to 0.62)	.302
Cardiorespiratory fitness (VO2max, mL/kg/min)^b^	0.99 (−0.41 to 2.39)	.164	0.33 (−0.92-1.57)	.606	0.23 (−0.90 to 1.35)	.694
Sedentary time (SWA, h/d)^a^	**−1.23 (−1.69 to −0.79)**	<**.001**	**−0.40 (−0.74 to −0.07)**	**.019**	**−0.64 (−0.99 to −0.28)**	<**.001**
Sleep (SWA, h/d)	0.01 (−0.32 to 0.35)	.945	−0.04 (−0.31 to 0.23)	.780	−0.24 (−0.51 to 0.04)	.08

*Abbreviations*: MVPA, moderate-to-vigorous intensity physical activity; β, unstandardized regression coefficients; MFI, Multidimensional Fatigue Inventory; EORTC QLQ-C30, European Organisation for Research and Treatment of Cancer; HADS, Hospital Anxiety and Depression scale; WHODAS, World Health Organization Disability Assessment Schedule; VO2max, maximal volume of oxygen uptake; SWA, SenseWear Armband mini.

*Note:* Four categories with different long-term MVPA patterns were created: “High & Increasing” (participants with high MVPA level immediately post-intervention and increased MVPA at 12-month follow-up), “High & Decreasing” (participants with high MVPA level immediately post-intervention and decreased MVPA at 12-month follow-up), “Low & Increasing” (participants with low MVPA level immediately post-intervention and increased MVPA at 12-month follow-up) and “Low & Decreasing” (participants with low MVPA level immediately post-intervention and decreased MVPA at 12-month follow-up). The category “Low & Decreasing” was used as reference in the analyses to test the hypothesis that high and/or increased physical activity after the end of an exercise intervention would be associated with improved health outcomes compared to those remaining less active. All models included immediately post-intervention value of the outcome to increase precision and were adjusted for age, gender and cancer treatment. Bold indicates p-value < .05.

a Higher scores indicate worse outcome.

b Higher scores indicate better outcome.

c For participants who reported working.

Compared to the participants in the “Low & Decreasing” category, those in the “High & Increasing” category reported significantly lower fatigue in 3 domains: general fatigue (β = −1.77, 95% CI [−3.08 to −0.46], *P* = .008), physical fatigue (β = −3.36, 95% CI [− 4.83 to −1.89], *P* < .001) and reduced activity (β = −1.58, 95% CI [−2.82 to −0.34], *P* = .012). They also reported higher HRQoL (β = 6.84, 95% CI [0.84 to −12.84], P = .026) and had less sedentary time at 12-month follow-up (β = −1.23, 95% CI [−1.69 to −0.79], *P* < .001) (Table 3).

Additionally, compared to the participants in the “Low & Decreasing” category, those in the “High & Decreasing” category reported significantly lower physical fatigue (β = −1.90, 95% CI [−3.09 to −0.71], *P* = .002), improved social participation (β= −1.43, 95% CI [−2.70 to −0.16], *P* = .027) and had less sedentary time at 12-month follow-up (β = −.40, 95% CI [−0.74 to −0.07], *P* < .019) (Table 3).

Finally, compared to the participants in the “Low & Decreasing” category, those in the “Low & Increasing” category had less sedentary time at 12-month follow-up (β = −.64, 95% CI [−0.99 to −0.28], *P* < .001) (Table 3).

## Discussion

In the present study, we investigated MVPA in cancer survivors who previously participated in an exercise intervention during curative cancer treatment. The aims were to assess the associations of (1) MVPA level at 12-months follow-up and (2) long-term MVPA patterns (from immediately post-intervention to 12-month follow-up) with different cancer-related health outcomes. We found that a daily increase of 30 minutes MVPA at 12-month follow-up was significantly associated with lower cancer-related fatigue, higher cardiorespiratory fitness, and less sedentary time at 12-month follow-up. Additionally, participants who had high MVPA levels immediately post-intervention and increased their MVPA levels at 12-month follow-up were those who had the greatest health benefits in terms of lower fatigue, higher HRQoL and less sedentary time.

As hypothesized, both higher MVPA level at 12-month follow-up and increased long-term MVPA patterns, were significantly associated with improved cancer-related health outcomes. However, some differences between the 2 physical activity measures regarding the observed associations are worth noting. For example, both MVPA level at 12-month follow-up and long-term MVPA patterns were significantly associated with lower fatigue, but it is the most physically active participants (those in the “High & Increasing” category) who reported clinically relevant lower scores in physical fatigue compared to the least physically active participants (those in the “Low & Decreasing” category), as the difference in score exceeded the minimal clinically importance of 2 points.^42^ Moreover, a strong positive association was found between long-term MVPA patterns and HRQoL for the most physically active participants. These results are in line with previous research, where breast cancer survivors who were more physically active 12-month^18^ and 24-month^17^ post-intervention consistently reported lower levels of fatigue and higher levels of HRQoL compared with those who were less physically active. Interestingly, MVPA level at 12-month follow-up was significantly associated with higher cardiorespiratory fitness while such an association was not found for long-term MVPA patterns. These results indicate that a daily increase of 30 minutes MVPA may improve cardiorespiratory fitness in cancer survivors. On the other hand, the absence of significant associations with long-term MVPA patterns could be explained by the already high levels of cardiorespiratory fitness in our study participants immediately post-intervention,^43,44^ making further progress at follow-up difficult to obtain.

Results regarding sedentary time indicate that both MVPA level at 12-month follow-up and long-term MVPA patterns were significantly and negatively associated with this outcome. Notably, compared to the category with the least physically active participants (those in the “Low & Decreasing” category), all the other categories were superior suggesting that high and/or increased MVPA levels after the end of an exercise intervention may reduce time spent sedentary in cancer survivors. This is in line with an RCT where breast cancer survivors randomized to a 12-week of MVPA both significantly increased MVPA and reduced sedentary time compared with the control group.^45^ It is important to note that individuals who meet the physical activity guidelines can still be highly sedentary^46^ (eg, an individual can be physically active 1 hour per day but still sit working on a computer 10 hours per day). Our results are of interest as sedentary time has emerged as a new risk factor for health and reducing this behavior may improve health outcomes in cancer survivors.^47^

In general, participants in the “High & Increasing” category was superior to the other groups for most cancer-related health outcomes. These results suggest that having high MVPA levels immediately post-intervention and increasing these levels during the follow-up period may be optimal for health outcomes in the long term in cancer survivors. These results also reinforce a possible dose-response relationship between MVPA levels and different cancer-related health outcomes and support advice within existing international physical activity guidelines that exceeding recommended physical activity levels is likely to provide additional health benefits in cancer survivors.^48^ Considering that the majority of cancer survivors do not reach the recommended levels, those results highlight the need for behavioral support with long-term effect on physical activity in cancer survivors. Such support could include the use of self-regulatory behavior change techniques (eg, goal-setting and self-monitoring), which have been proven effective in promoting long-term physical activity post-intervention in this population.^14^

There are numerous strengths in this study. First, we extend current knowledge through (1) demonstrating the potential long-term benefits of increasing MVPA levels post-intervention for different cancer-related health outcomes and (2) providing evidence of such associations when physical activity levels are objectively assessed. Further, our findings add to the growing evidence that a dose-response relationship between MVPA levels and health benefits in cancer survivors exists. Strengths in this study also include a large sample, the longitudinal design and long-term follow-up. However, our study is not without limitations including the large number of participants lost to follow-up and the homogeneous study sample (ie, mainly women with breast cancer, highly educated and physically active when entering the study), limiting the generalizability of our results. Additionally, although our study design does not allow to determine the direction of causality, adjusting for several confounders and examining both MVPA levels at 12-month follow-up and long-term MVPA patterns strengthens our conclusions about the observed associations. Our multidimensional approach, combining absolute levels of physical activity (ie, MVPA level) with a relative measure of physical activity (ie, long-term MVPA patterns) also provides an accurate and well-rounded picture of cancer survivors’ long-term physical activity status.^23^ Finally, long-term MVPA patterns was determined based on the sample’s median of MVPA immediately post-intervention (ie, high MVPA level ≥ median and low MVPA level <median) and the changes between 2 measurement points (ie, immediately post-intervention and 12-month follow-up). This method for categorization has 2 implications. First, participants were considered as having a low MVPA level if below the median although a majority was quite physically active. Second, because we did not assess MVPA between those 2 measurements, we could not determine how physically active participants were during the 12-month period. Some participants may have changed their MVPA level very late during this period, which is not reflected in our classification. However, although the results need to be interpreted in the light of these limitations, our classification provides a unique and detailed depiction of physical activity patterns in our study sample.

## Conclusion

Our results suggest that long-term physical activity is essential for improving health outcomes post-intervention in cancer survivors, even among those with high MVPA levels. Cancer survivors, including those who reach recommended MVPA levels, should therefore be encouraged to maintain or increase MVPA post-intervention for additional health benefits.

## Supplemental Material

sj-docx-1-ict-10.1177_15347354231178869 – Supplemental material for The Role of Long-Term Physical Activity in Relation to Cancer-Related Health Outcomes: A 12-Month Follow-up of the Phys-Can RCT Click here for additional data file. Supplemental material, sj-docx-1-ict-10.1177_15347354231178869 for The Role of Long-Term Physical Activity in Relation to Cancer-Related Health Outcomes: A 12-Month Follow-up of the Phys-Can RCT by Anne-Sophie Mazzoni, Ann Christin Helgesen Bjørke, Andreas Stenling, Sussanne Börjeson, Katarina Sjövall, Sveinung Berntsen, Ingrid Demmelmaier and Karin Nordin in Integrative Cancer Therapies

sj-docx-2-ict-10.1177_15347354231178869 – Supplemental material for The Role of Long-Term Physical Activity in Relation to Cancer-Related Health Outcomes: A 12-Month Follow-up of the Phys-Can RCT Click here for additional data file. Supplemental material, sj-docx-2-ict-10.1177_15347354231178869 for The Role of Long-Term Physical Activity in Relation to Cancer-Related Health Outcomes: A 12-Month Follow-up of the Phys-Can RCT by Anne-Sophie Mazzoni, Ann Christin Helgesen Bjørke, Andreas Stenling, Sussanne Börjeson, Katarina Sjövall, Sveinung Berntsen, Ingrid Demmelmaier and Karin Nordin in Integrative Cancer Therapies
