# Supplementary material for: The Role of Long-Term Physical Activity in Relation to Cancer-Related Health Outcomes: A 12-Month Follow-up of the Phys-Can RCT
Source: Integr Cancer Ther. 2023 Jun 26;22:15347354231178869. doi: 10.1177/15347354231178869 (PMC10331773; doi:10.1177/15347354231178869)
Supplement: sj-docx-1-ict-10.1177_15347354231178869 – Supplemental material for The Role of Long-Term Physical Activity in Relation to Cancer-Related Health Outcomes: A 12-Month Follow-up of the Phys-Can RCT [file sj-docx-1-ict-10.1177_15347354231178869.docx]

| **Suppl. Table 1.** Descriptive data for objectively assessed physical activity immediately post-intervention and at 12-month follow-up. | | | | | | | | | | | |  |
| --- | --- | --- | --- | --- | --- | --- | --- | --- | --- | --- | --- | --- |
|  | **All**  **(*n*=353)** | |  | **Long-term MVPA patterns**^a^  **(*n*=316)** | | | | | | | | |
|  |  | |  | **High & Increasing**  **(*n*=46)** | | **High & Decreasing**  **(*n*=112)** | | **Low & Increasing**  **(*n*=91)** | | **Low & Decreasing**  **(*n*=67)** | | |
|  | **Mean (*SD*)** | **Median (IQR)** |  | **Mean (*SD*)** | **Median (IQR)** | **Mean (*SD*)** | **Median (IQR)** | **Mean (*SD*)** | **Median (IQR)** | **Mean (*SD*)** | **Median (IQR)** | |
| **MVPA, post-intervention (min/week)** | 520 (360) | 464 (386) |  | 700 (238) | 598 (295) | 807 (367) | 692 (397) | 238 (122) | 213 (198) | 301 (116) | 333 (213) | |
| **MVPA, 12-month follow-up (min/week)** | 484 (327) | 433 (383) |  | 875 (338) | 786 (495) | 552 (306) | 505 (301) | 420 (240) | 387 (272) | 203 (112) | 206 (178) | |
| **Change in MVPA between post-intervention and 12-month follow-up (%)** | +20 (153) | -8 (65) |  | +24 (20) | +18 (30) | -31 (21) | -29 (32) | +119 (255) | +65 (94) | -34 (23) | -28 (35) | |
|  |  |  |  |  |  |  |  |  |  |  |  | |
| *Abbreviations*: MVPA: moderate-to-vigorous intensity physical activity, 12-month follow-up: 12 months post-intervention; SD: standard deviation; IQR: interquartile range. *Note*: ^a^Four categories with different long-term MVPA patterns were created: “High & Increasing” (participants with high MVPA level immediately post-intervention and increased MVPA at 12-month follow-up), “High & Decreasing” (participants with high MVPA level immediately post-intervention and decreased MVPA at 12-month follow-up), “Low & Increasing” (participants with low MVPA level immediately post-intervention and increased MVPA at 12-month follow-up) and “Low & Decreasing” (participants with low MVPA level immediately post-intervention and decreased MVPA at 12-month follow-up). | | | | | | | | | | | |  |
