# Supplementary material for: The Role of Long-Term Physical Activity in Relation to Cancer-Related Health Outcomes: A 12-Month Follow-up of the Phys-Can RCT
Source: Integr Cancer Ther. 2023 Jun 26;22:15347354231178869. doi: 10.1177/15347354231178869 (PMC10331773; doi:10.1177/15347354231178869)
Supplement: sj-docx-2-ict-10.1177_15347354231178869 – Supplemental material for The Role of Long-Term Physical Activity in Relation to Cancer-Related Health Outcomes: A 12-Month Follow-up of the Phys-Can RCT [file sj-docx-2-ict-10.1177_15347354231178869.docx]

**Suppl. Table 2.** Descriptive data for cancer-related health outcome measures immediately post-intervention and at 12-month follow-up for patients with data available.

|  | **Post-intervention** | |  | **12-month follow-up** | |
| --- | --- | --- | --- | --- | --- |
|  | ***n*** | **Mean (*SD*)** |  | ***n*** | **Mean (*SD*)** |
| **Cancer-related fatigue (MFI, 4-20)**^a^ |  |  |  |  |  |
| General fatigue |  |  |  |  |  |
| All | 330 | 10.4 (4.3) |  | 331 | 10.6 (4.4) |
| High & Increasing | 44 | 9.8 (4.2) |  | 45 | 9.1 (4.3) |
| High & Decreasing | 108 | 9.5 (4.0) |  | 104 | 9.8 (4.2) |
| Low & Increasing | 84 | 10.6 (4.7) |  | 81 | 11.1 (4.5) |
| Low & Decreasing | 65 | 11.2 (4.1) |  | 64 | 11.7 (4.3) |
| Physical fatigue |  |  |  |  |  |
| All | 332 | 8.9 (4.1) |  | 334 | 10.1 (4.5) |
| High & Increasing | 46 | 7.8 (3.7) |  | 44 | 7.6 (3.7) |
| High & Decreasing | 108 | 7.7 (3.2) |  | 107 | 9.0 (4.0) |
| Low & Increasing | 84 | 9.8 (4.6) |  | 81 | 11.5 (4.5) |
| Low & Decreasing | 64 | 9.5 (4.1) |  | 65 | 11.6 (4.5) |
| Reduced activity |  |  |  |  |  |
| All | 331 | 9.5 (4.1) |  | 327 | 9.6 (3.9) |
| High & Increasing | 46 | 8.5 (4.1) |  | 43 | 7.7 (3.7) |
| High & Decreasing | 110 | 8.6 (3.7) |  | 103 | 9.1 (3.5) |
| Low & Increasing | 82 | 10.4 (4.4) |  | 80 | 10.4 (4.3) |
| Low & Decreasing | 64 | 10.1 (4.2) |  | 64 | 10.2 (4.0) |
| Reduced motivation |  |  |  |  |  |
| All | 331 | 7.9 (3.3) |  | 335 | 7.9 (3.3) |
| High & Increasing | 46 | 7.4 (3.4) |  | 45 | 7.1 (3.2) |
| High & Decreasing | 108 | 7.6 (3.0) |  | 107 | 7.3 (2.9) |
| Low & Increasing | 83 | 8.0 (3.6) |  | 80 | 8.3 (3.5) |
| Low & Decreasing | 64 | 8.0 (3.4) |  | 66 | 8.1 (3.4) |
| Mental fatigue |  |  |  |  |  |
| All | 329 | 8.8 (4.0) |  | 332 | 8.8 (3.8) |
| High & Increasing | 46 | 8.2 (4.2) |  | 44 | 8.3 (4.1) |
| High & Decreasing | 109 | 8.3 (3.7) |  | 105 | 8.2 (3.2) |
| Low & Increasing | 81 | 9.4 (4.1) |  | 82 | 9.2 (4.0) |
| Low & Decreasing | 64 | 8.8 (3.8) |  | 65 | 8.8 (4.1) |
| **HRQoL (EORTC QLQ-C30, 0-100)** ^b^ |  |  |  |  |  |
| All | 338 | 76.3 (17.4) |  | 342 | 74.8 (18.1) |
| High & Increasing | 46 | 75.2 (19.0) |  | 45 | 77.4 (19.8) |
| High & Decreasing | 110 | 81.4 (14.1) |  | 109 | 78.2 (15.1) |
| Low & Increasing | 87 | 73.0 (18.7) |  | 84 | 72.9 (18.1) |
| Low & Decreasing | 65 | 74.5 (17.7) |  | 67 | 70.4 (20.1) |
| **Anxiety and depression (HADS, 0-21)**^a^ |  |  |  |  |  |
| Anxiety |  |  |  |  |  |
| All | 338 | 3.8 (3.6) |  | 343 | 3.9 (3.6) |
| High & Increasing | 46 | 4.5 (4.1) |  | 45 | 4.2 (4.2) |
| High & Decreasing | 110 | 3.3 (3.1) |  | 110 | 3.6 (3.5) |
| Low & Increasing | 87 | 3.9 (3.8) |  | 84 | 3.7 (3.6) |
| Low & Decreasing | 65 | 3.7 (3.5) |  | 67 | 3.8 (3.5) |
| Depression |  |  |  |  |  |
| All | 338 | 2.6 (2.9) |  | 343 | 2.7 (2.9) |
| High & Increasing | 46 | 2.9 (3.6) |  | 45 | 2.3 (3.3) |
| High & Decreasing | 110 | 2.1 (2.5) |  | 110 | 2.2 (2.4) |
| Low & Increasing | 87 | 2.9 (2.9) |  | 84 | 3.2 (3.2) |
| Low & Decreasing | 65 | 2.8 (3.1) |  | 67 | 2.6 (3.1) |
| **Functioning in daily life (WHODAS)** ^a^ |  |  |  |  |  |
| Work subscale (0-16) ^c^ |  |  |  |  |  |
| All | 148 | 3.5 (3.6) |  | 220 | 2.6 (3.3) |
| High & Increasing | 16 | 3.9 (3.8) |  | 34 | 2.8 (3.5) |
| High & Decreasing | 51 | 2.9 (3.5) |  | 73 | 1.7 (2.8) |
| Low & Increasing | 38 | 4.0 (3.6) |  | 50 | 2.5 (3.1) |
| Low & Decreasing | 35 | 3.5 (3.7) |  | 43 | 3.4 (3.7) |
| Social Participation subscale (0-32) |  |  |  |  |  |
| All | 307 | 5.7 (4.6) |  | 340 | 4.8 (4.8) |
| High & Increasing | 41 | 6.7 (5.2) |  | 46 | 5.3 (5.5) |
| High & Decreasing | 99 | 4.9 (4.4) |  | 109 | 3.8 (4.4) |
| Low & Increasing | 78 | 5.6 (4.5) |  | 82 | 4.9 (4.7) |
| Low & Decreasing | 63 | 5.8 (4.9) |  | 67 | 5.5 (5.1) |
| **Cardiorespiratory fitness (VO2max, mL/kg/min)** ^b^ |  |  |  |  |  |
| All | 317 | 30.2 (7.0) |  | 269 | 28.8 (7.2) |
| High & Increasing | 43 | 34.0 (7.4) |  | 42 | 32.9 (7.8) |
| High & Decreasing | 108 | 31.7 (6.4) |  | 85 | 29.8 (6.6) |
| Low & Increasing | 80 | 27.9 (6.6) |  | 73 | 26.4 (6.0) |
| Low & Decreasing | 60 | 27.8 (5.5) |  | 45 | 26.3 (5.9) |
| **Sedentary time (SWA, h/day)**^a^ |  |  |  |  |  |
| All | 316 | 10.9 (2.0) |  | 353 | 11.1 (1.9) |
| High & Increasing | 46 | 9.8 (1.8) |  | 46 | 9.5 (1.9) |
| High & Decreasing | 112 | 10.2 (1.8) |  | 112 | 10.8 (1.7) |
| Low & Increasing | 91 | 11.9 (2.1) |  | 91 | 11.7 (2.1) |
| Low & Decreasing | 67 | 11.6 (1.6) |  | 67 | 11.9 (1.6) |
| **Sleep (SWA, h/day)** |  |  |  |  |  |
| All | 316 | 7.1 (1.1) |  | 353 | 7.1 (1.1) |
| High & Increasing | 46 | 7.2 (1.1) |  | 46 | 7.2 (1.0) |
| High & Decreasing | 112 | 7.0 (0.7) |  | 112 | 7.1 (0.9) |
| Low & Increasing | 91 | 7.1 (1.6) |  | 91 | 7.0 (1.4) |
| Low & Decreasing | 67 | 7.1 (0.9) |  | 67 | 7.3 (1.0) |
| *Abbreviations*: 12-month follow-up: 12 months post-intervention; SD: standard deviation; MFI: Multidimensional Fatigue Inventory; EORTC QLQ-C30: European Organisation for Research and Treatment of Cancer; HADS: Hospital Anxiety and Depression scale; WHODAS: World Health Organization Disability Assessment Schedule; VO2max: maximal volume of oxygen uptake; SWA: SenseWear Armband mini. *Note*: ^a^ Higher scores indicate worse outcome, ^b^ Higher scores indicate better outcome, ^c^ For participants who reported working. “High & Increasing”: participants with high moderate-to-vigorous intensity physical activity (MVPA) level immediately post-intervention and increased MVPA at 12-month follow-up),“High & Decreasing”: participants with high MVPA level immediately post-intervention and decreased MVPA at 12-month follow-up, “Low & Increasing”: participants with low MVPA level immediately post-intervention and increased MVPA at 12-month follow-up and “Low & Decreasing”: participants with low MVPA level immediately post-intervention and decreased MVPA at 12-month follow-up. | | | | | |
